# Effects and Mechanisms of Probiotics, Prebiotics, Synbiotics, and Postbiotics on Metabolic Diseases Targeting Gut Microbiota: A Narrative Review

**DOI:** 10.3390/nu13093211

**Published:** 2021-09-15

**Authors:** Hang-Yu Li, Dan-Dan Zhou, Ren-You Gan, Si-Yu Huang, Cai-Ning Zhao, Ao Shang, Xiao-Yu Xu, Hua-Bin Li

**Affiliations:** 1 Guangdong Provincial Key Laboratory of Food, Nutrition and Health, Department of Nutrition, School of Public Health, Sun Yat-Sen University, Guangzhou 510080, China; lihy277@mail2.sysu.edu.cn (H.-Y.L.); zhoudd6@mail2.sysu.edu.cn (D.-D.Z.); huangsy9@mail2.sysu.edu.cn (S.-Y.H.); shangao@mail2.sysu.edu.cn (A.S.); xuxy53@mail2.sysu.edu.cn (X.-Y.X.); 2 Research Center for Plants and Human Health, Institute of Urban Agriculture, Chinese Academy of Agricultural Sciences, Chengdu 610213, China; ganrenyou@yahoo.com; 3 Key Laboratory of Coarse Cereal Processing (Ministry of Agriculture and Rural Affairs), School of Food and Biological Engineering, Chengdu University, Chengdu 610106, China; 4 Department of Clinical Oncology, Li Ka Shing Faculty of Medicine, The University of Hong Kong, Hong Kong 999077, China; zhaocn@connect.hku.hk; 5 School of Chinese Medicine, Li Ka Shing Faculty of Medicine, The University of Hong Kong, Hong Kong 999077, China

**Keywords:** probiotics, prebiotics, synbiotics, postbiotics, gut microbiota, obesity, type 2 diabetes mellitus, gestational diabetes mellitus, metabolic diseases

## Abstract

Metabolic diseases are serious threats to public health and related to gut microbiota. Probiotics, prebiotics, synbiotics, and postbiotics (PPSP) are powerful regulators of gut microbiota, thus possessing prospects for preventing metabolic diseases. Therefore, the effects and mechanisms of PPSP on metabolic diseases targeting gut microbiota are worth discussing and clarifying. Generally, PPSP benefit metabolic diseases management, especially obesity and type 2 diabetes mellitus. The underlying gut microbial-related mechanisms are mainly the modulation of gut microbiota composition, regulation of gut microbial metabolites, and improvement of intestinal barrier function. Moreover, clinical trials showed the benefits of PPSP on patients with metabolic diseases, while the clinical strategies for gestational diabetes mellitus, optimal formula of synbiotics and health benefits of postbiotics need further study. This review fully summarizes the relationship between probiotics, prebiotics, synbiotics, postbiotics, and metabolic diseases, presents promising results and the one in dispute, and especially attention is paid to illustrates potential mechanisms and clinical effects, which could contribute to the next research and development of PPSP.

## 1. Introduction

Metabolic syndrome is a cluster of metabolic disorders, manifested as dyslipidemia, hyperglycemia, insulin resistance, oxidative stress, inflammation, hypertension, and neurodegeneration, which is associated with metabolic diseases, such as obesity, diabetes mellitus, non-alcoholic fatty liver disease (NAFLD), and osteoarthritis [1,2]. Moreover, different metabolic diseases are often associated with each other, for example, obesity is a risk factor of type 2 diabetes mellitus and excess body weight also contributes to the development of NAFLD [3]. Recent studies have found out that the imbalance of gut microbiota is crucial to metabolic diseases [4,5]. Therefore, the regulation of gut microbiota could be a promising way out of this situation [6]. On the one hand, it is generally recognized that gut microbial homeostasis itself is a vital part of the whole metabolic system so that the regulation of beneficial and conditioned pathogenic bacteria is one of the most direct mechanisms to improve metabolic function [7]. On the other hand, gut microbiota continuously excretes bioactive molecules in the bowel lumen and some of them may translocate into the circulation and further make an influence on the metabolic process as specific ligands [8]. During the process, intestinal integrity plays an important role by blocking the harmful bacterial metabolites transferred into the circulation [9].

Probiotics are a series of beneficial microorganisms, and prebiotics are traditionally non-digestible food ingredients that selectively stimulate the growth and activity of a limited number of bacteria in the digestive tract [10]. Recently, there is an emerging trend to explore whether synbiotics (a combination of prebiotics and probiotics) and postbiotics (inanimate microorganisms and/or their components that confers a health benefit on the host) possess good biological activities for the prevention of metabolic diseases [11,12,13]. According to the results from experimental studies and clinical trials, probiotics, prebiotics, synbiotics, and postbiotics (PPSP) have shown alleviated effects on obesity, type 2 diabetes mellitus, and other metabolic diseases in most cases, but the situation could be uncertain when the patients are during pregnancy [14]. In this review, we summarize the results from recent studies (Web of Science within recent five years) to comprehensively exhibit the bioactive effects, potential mechanisms, and clinical action of PPSP on metabolic diseases.

## 2. Bioactive Effects of Probiotics, Prebiotics, Synbiotics, and Postbiotics on Metabolic Diseases

### 2.1. Obesity

Obesity is intimately related to gut microbiota, and probiotic strains possess beneficial effects on attenuating obesity, such as *Lactobacillus pentosus* GSSK2 and *Lactobacillus plantarum* GS26A [15]. Several studies showed that the anti-obesity effects of probiotics were different between the lyophilized bacteria and alive bacteria, as well as the single-strain and multi-strain [16,17]. Several studies highlighted that the lyophilized *Lactobacillus casei* IMVB-7280 possessed stronger anti-obesity property than lyophilized *Bifidobacterium animalis* VKB and VKL strains [16]. Moreover, the alive multi-strains involving Acetobacter, *Bifidobacterium*, *Lactobacillus*, and *Propionibacterium* could more effectively improve obesity, insulin resistance, pro-inflammatory cytokines production, and adiponectin level when compared with the lyophilized single-strain or multi-strains [18]. Besides, diverse evidence showed that prebiotics (like polyphenols) could impact the progression of obesity via the regulation of gut microbiota [19,20]. Owing to the promising application value of prebiotics in the management of metabolic diseases, researchers keep modifying the strategies of food processing to enhance the therapeutic effects [21,22]. For example, the anti-obesity effect of resistant starch could be elevated after modifying with the amylosucrase from *Deinococcus geothermalis* [23]. Moreover, synbiotics containing *Bifidobacterium lactis*, *Lactobacillus rhammnosus*, and oligofructose-enriched inulin could more effectively regulate the intestinal microenvironment than single components [10,24]. What’s more, an in vivo study based on resveratrol-fed mice implied that postbiotics could be the reason why resveratrol was beneficial to anti-obesity, but further clinical research was needed to provide more reliable evidence [25].

### 2.2. Type 2 and Gestational Diabetes Mellitus

Fermented foods like yogurt were beneficial to type 2 diabetic patients, especially those who were refractory to conventional therapy [26,27]. A prospective study on 8574 adults suggested that yogurt consumption was inversely associated with the risk of type 2 diabetes mellitus (hazard ratio (HR): 0.73; 95% confidence interval (CI): 0.61, 0.88) [28]. However, the side effects of sugar-sweetened yogurt have caused concern [29]. Because of the promising beneficial effects of probiotics on type 2 diabetes mellitus, researchers keep optimizing the applicational strategy, for example, the utilization of the monk fruit extract as a novel sweetener in yogurt could more effectively improve serum lipid level than yogurt alone, and simultaneously avoid the health risk caused by sugar [30]. Moreover, several prebiotic foods like *Lactobacillus plantarum*-fermented *Momordica charantia* juice possessed a better anti-diabetic effect than the native one [31]. Furthermore, because the treatment of type 2 diabetes mellitus is often accompanied by the regulation of gut bacterial metabolites, postbiotics could be an alternative therapeutic agent for probiotics, prebiotics, and fecal microbiota transplant [32]. However, other studies implied that the consumption of synbiotic foods only revealed limited mitigation in diabetes mellitus, especially gestational diabetes mellitus [33,34]. For example, adding extra fish oil into probiotic supplements containing *Bifidobacterium lactis* and *Lactobacillus rhamnosus* HN001 failed to reduce the risk of gestational diabetes mellitus whether combined as synbiotics or used as single agents [35].

### 2.3. Other Metabolic Diseases

The utilization of probiotics has been recognized as a potential treatment for other metabolic diseases like NAFLD [36]. Moreover, a cross-sectional study with 26,016 subjects reported that the intake of prebiotics, such as dietary fiber, phytochemicals, and complex carbohydrates, was inversely associated with the risk of metabolic disorders [37]. Furthermore, gut microbiota played a role in metabolic-related diseases especially when accompanied by low-grade chronic inflammation [38,39,40]. Prebiotics like polysaccharides from *Cyclocarya paliurus* could improve metabolic function and chronic inflammation by increasing *Bacteroidetes* abundance and downregulating Toll-like receptor 4-mitogen-activated protein kinase (TLR4-MAPK) signaling pathway [41]. However, the abundances of *Bacteroides* and *Parabacteroides* were decreased in obese mice with low-grade inflammation after receiving unsaturated alginate oligosaccharides [42]. Besides, the oral administration of glucose solution with probiotics could improve intestinal barrier function and inflammation in patients after colorectal cancer surgery [43]. Several studies highlighted that the synergistic effects of probiotics and prebiotics on dyslipidemia and hypercholesterolemia remained a mystery [44]. The co-administration of probiotics and natural prebiotic food like *Agaricus bisporus* mushroom revealed the same curative effect on dyslipidemia as each agent alone [44]. Likely, the combination of probiotics (*Lactobacillus acidophilus* La-5) and prebiotics (inulin oligofructose) did not show a better therapeutic effect on metabolic-related diseases compared with the prebiotics or probiotics alone [45]. 

In short, PPSP could alleviate metabolic diseases, and the co-intervention of multiple alive bacteria strains could be a promising way of probiotics application. Moreover, the enzyme-modification and probiotic-fermentation could effectively enhance the benefits of prebiotics. Furthermore, the effects of *Bacteroidetes* in metabolic diseases accompanied with low-grade inflammation need further investigation.

## 3. Mechanisms of Probiotics, Prebiotics, Synbiotics, and Postbiotics on Metabolic Diseases by Targeting Gut Microbiota

The intervention of probiotics (like *Bifidobacterium* and *Lactobacillus*), prebiotics (like inulin oligofructose and other polysaccharides), synbiotics (consist of probiotic strains and prebiotic foods), and postbiotics (like short-chain fatty acids (SCFAs) and muramyl dipeptide) could make an important influence on metabolic function. The recent studies showed that the modulation of gut microbiota composition, regulation of gut microbial metabolites, as well as improvement of the intestinal barrier function were three major mechanisms of PPSP on regulating metabolic diseases, which would be discussed below.

### 3.1. The Modulation of Gut Microbiota Composition

Probiotics have been proposed as a suitable strategy for preventing metabolic diseases [46]. The mixture of probiotics containing *Bifidobacterium lactis* LMG P-28149 and *Lactobacillus rhamnosus* LMG S-28148 could modulation the composition of obesity-related gut microbiota and restore *Akkermansia muciniphila* and *Rikenellaceae* abundances while reducing *Lactobacillaceae* abundance [47]. Besides, other metabolic-related diseases like inflammatory bowel disease were associated with digestive system dysfunction and gut microbiota dysbiosis, manifested as the less abundances of *Bifidobacteria* and *Lactobacillus*, as well as a higher abundance of *Escherichia coli* [48]. Moreover, gut microbiota is considered as a trigger for the metabolic inflammation in obesity and type 2 diabetes mellitus, and the administration of *Lactobacillus reuteri* could improve metabolic function by inhibiting the growth of harmful bacteria (like *Yersinia enterocolitica*) and improving tetrathionate metabolism in TLR1-deficient mice with intestinal inflammation [49,50]. Furthermore, the intake of *Lactobacillus casei* could prevent metabolic-related hypertension in perinatal rats by decreasing the *Firmicutes* to *Bacteroidetes* ratio and the expression of angiotensin-converting enzyme (ACE), while increasing *Akkermansia* and *Lactobacillus* abundances [51]. In addition, an in vivo study with male Swiss albino mice reported that the probiotic-fermented milk containing *Lactobacillus reuteri* LR6 could improve protein-energy malnutrition and immunological function in mice with increasing *Bifidobacteria*, *Firmicutes*, and *Lactobacilli* abundances [52]. 

The healthy influence of gut microbiota was started on the early stage of life, and the experimental studies showed that prebiotic dextrin from maize starch and lentil-based diet could promote the growth of *Actinobacteria* and *Bacteroidetes*, while decrease *Firmicutes* abundance, thus might be beneficial to overweight and obese children [53,54,55]. Moreover, resistant dextrin from wheat and corn starch could improve lipid and glucose metabolism in insulin resistance mice by increasing *Akkermansia* and *Prevotella* abundances, followed by upregulating the insulin receptor substrate 1 (IRS1)-protein kinase B (Akt)-glucose transporter 2 (GLUT2) signaling pathway as well as upregulating the sirtuin 1 (SIRT1)-adenosine monophosphate kinase (AMPK)-peroxisome proliferators activated receptor α (PPARα)-carnitine palmitoyltransferase 1α (CPR1α) signaling pathway [56]. Furthermore, the supplementation of whole garlic improved serum lipid profile, liver function, and insulin resistance in dyslipidemia mice, with increasing *Lachnospiraceae* while reducing *Prevotella* abundances [57]. Inulin was a common prebiotic, and the in vivo studies showed that the intake of inulin oligofructose improved obesity, glycemic dysregulations, and the blood-brain-barrier integrity, with decreasing the *Firmicutes* to *Bacteroidetes* ratio while increasing *Bifidobacterium* abundance [58,59]. Additionally, the pregnant rats which received oligofructose had a lower obesity risk in their offspring, with higher abundances of *Bifidobacterium* and *Collinsella* [60]. Besides, fucoidan polysaccharides could improve gut microbial diversity with increasing the abundance of *Bacteroidetes* and *Proteobacteria*, enhancing the bile salt hydrolase activity of *Lactobacillus casei* DM8121, as well as upregulating the expression of hepatic cholesterol 7-α hydroxylase, while the abundances of *Actinobacteria* and *Firmicutes* were decreased [61,62].

Synbiotics were considered as a new frontier in obesity prevention, and the omega-3 fatty acids with a mixture of alive probiotics containing *Bifidobacterium*, *Lactobacillus*, *Lactococcus*, and *Propionibacterium* showed a more pronounced reduction in hepatic steatosis and lipid accumulation compared to probiotics alone [63,64]. Moreover, the oral supplements of combined *Bacillus licheniformis* and xylo-oligosaccharides could more effectively improve body weight gain and lipid metabolism in obese rats with decreasing the abundances of *Desulfovibrionaceae* and *Ruminococcaceae* [65]. In addition, a mixture of *Lactobacillus plantarum* PMO 08 with chia seeds revealed a synergistic anti-obesity effect on obese mice, and led to a more favorable intestinal microenvironment for *Lactobacillus plantarum* growth [66].

Butyrate was a typical postbiotics produced from gut microbiota, and it could alleviate intestinal inflammation induce by *Citrobacter rodentium*, and result in increasing *Lachnospiraceae* and *Proteobacteria* as well as decreasing *Clostridiaceae* abundances in mice [67]. Moreover, butyrate administration could ameliorate NAFLD and increase the abundances of butyric-produced bacteria such as *Blautia*, *Christensenellaceae*, and *Lactobacillus* in return [68]. So, there could be a bi-directional regulation and positive feedback action between the supplement of postbiotics like butyrate and the growth of related bacteria [69].

**Figure 1:**
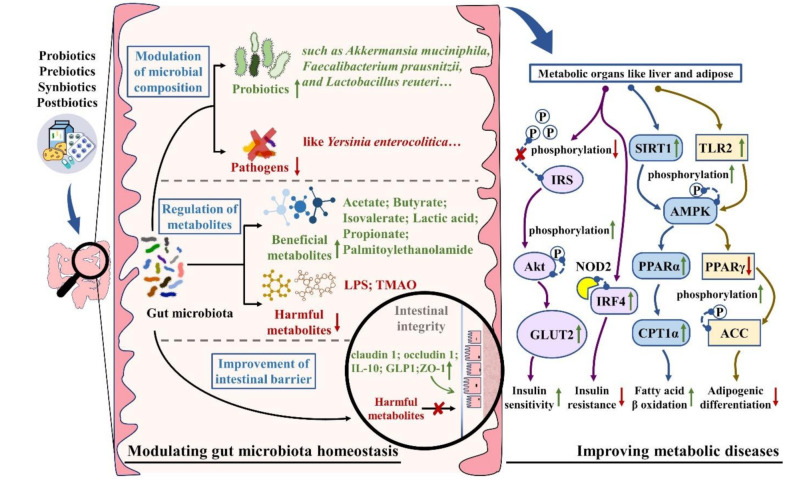
Mechanisms of PPSP on metabolic diseases targeting gut microbiota. PPSP directly alleviate metabolic diseases by regulating the abundances of beneficial and harmful bacteria. Besides, PPSP indirectly ameliorate metabolic diseases by promoting the production of acetate, propionate, butyrate, isovalerate, lactic acid, and palmitoylethanolamide, while suppressing the production of LPS, TMAO and reducing the bile acid pools. Moreover, the improvement of intestinal barrier function played an important role in attenuating metabolic diseases, with upregulating claudin 1, GLP1, IL-10, occludin 1, and ZO-1 expressions. These mechanisms collectively improve the whole metabolic system by targeting several pivotal molecular, such as upregulating IRS1-Akt-GLUT2, SIRT1-AMPK-PPARα-CPR1α, TLR2-PPARγ-AMPK, and NOD-IRF4 signaling pathways. Abbreviation: ACC, acetyl-CoA carboxylase; Akt, protein kinase B; AMPK, adenosine monophosphate kinase; CPR1α, carnitine palmitoyltransferase 1α; GLUT2, glucose transporter 2; IRF4, interferon regulatory factor 4; IRS1, insulin receptor substrate 1; LPS, lipopolysaccharide; NOD2, oligomerization domain-containing protein 2; PPARα/γ, peroxisome proliferators activated receptor α/γ; SIRT1, sirtuin 1; TLR2, Toll-like receptor 2; TMAO, trimethylamine-n-oxide; ZO-1, zonula occludens-1.

In general, PPSP could regulate metabolic diseases by regulating the abundances of *Akkermansia*, *Bacteroidetes*, *Blautia*, *Bifidobacteria*, *Bifidobacterium*, *Collinsella*, *Christensenellaceae*, *Desulfovibrionaceae*, *Lachnospiraceae*, *Lactobacillus*, *Proteobacteria*, *Rikenellaceae*, and *Ruminococcaceae*, while decreasing the abundances of *Clostridiaceae*, *Firmicutes*, *Lactobacillaceae*, and *Yersinia* (Figure 1). Meanwhile, the modulation of gut bacteria composition was accompanied by upregulating IRS1-Akt-GLUT2 and SIRT1-AMPK-PPARα-CPR1α signaling pathways while downregulating the mRNA expression of ACE, as well as improving tetrathionate metabolism, bile salt hydrolase activity, and hepatic cholesterol 7-alpha hydroxylase expression (Table 1). 

### 3.2. The Regulation of Gut Microbial Metabolites

Several gut microbial metabolites could indirectly influence host metabolism by interacting with several key proteins involved in metabolic-related signaling pathways, such as G-protein coupled receptor (GPR), AMPK, and PPARγ [92,93]. The in vitro study showed that the mixture of *Bifidobacterium lactis* and *Lactobacillus salivarius* could promote the production of butyrate and propionate in the fermentation system [47]. Moreover, after comparing 23 bacteria strains, an experimental study revealed that *Bifidobacterium longum* PI10 and *Ligilactobacillus salivarius* PI2 could restrict lipid accumulation in adipocytes [70]. Furthermore, the *Lactobacillus plantarum* NK3 and *Bifidobacterium longum* NK49 could mitigate obesity and osteoporosis in mice by suppressing the production of lipopolysaccharide (LPS) from gut microbiota and downregulating nuclear factor kappa B (NF-κB)-linked tumor necrosis factor α (TNF-α) expression [71]. Additionally, probiotics containing *Bifidobacterium breve* CECT7263 and *Lactobacillus fermentum* CECT5716 ameliorated metabolic-related hypertension in rats by increasing the abundance of butyrate-related bacteria, and elevating the level of butyrate in plasma while reducing the production of LPS [73]. What’s more, the engineered *Lactobacillus paracasei* could produce metabolites like palmitoylethanolamide to maintain normal intestinal function in mice [74].

The intake of prebiotics could regulate the production of gut microbial metabolites like SCFAs and bile acids thus influencing the metabolic process [94]. It has been reported that the administration of isomaltodextrin was positively related to the concentrations of acetic and butyric acids in mice with glucometabolic disorder [38]. Moreover, an in vivo study revealed that the intervention of dietary fiber from oat and rye brans improved body weight, glucose metabolism, hepatic inflammation, and SCFAs production in mice, accompanied with regulating bile acids and tryptophan-serotonin metabolism [77]. Furthermore, the amylosucrase-modified chestnut starch could ameliorate obesity in mice by upregulating the SCFAs-GPR43-mediated signaling pathway [23]. Besides, inulin and fructan from *Agave salmiana* could be beneficial to prevent metabolic syndrome and related hypertension by regulating gut microbial metabolites, manifested as increasing the levels of acetic, propionic, butyric, and lactic acids while decreasing the level of trimethylamine-n-oxide (TMAO) [51,75,78]. In addition, galacto-oligosaccharides could inhibit the progression of obesity and insulin resistance in mice with increasing intestinal glucagon-like peptide 1 (GLP1) expression while decreasing fecal bile acid excretion [76]. 

The synbiotic intervention also revealed regulatory function in gut microbial metabolites production, and the combination of *Bifidobacterium lactis*, *Lactobacillus paracasei* DSM 4633, and oat β-glucan could inhibit body weight gain and metabolic complications in obese mice with restoring the fecal levels of acetate, propionate, and butyrate, as well as reducing the bile acid pools [80]. Besides, synbiotics containing *Clostridium butyricum* and corn bran could decrease the abundances of pathogens while promoting the growth of acetate-produced bacteria, which further lead to an increase of acetate and isovalerate [84]. Moreover, synbiotics containing *Lactobacillus paracasei* N1115 and fructo-oligosaccharides alleviate NAFLD in mice, resulting in improving lipid metabolism with inhibiting the production of LPS, as well as downregulating related molecular targets like TLR4 and NF-κB [82]. Furthermore, the synbiotics containing *Bifidobacterium bifidum* V, *Lactobacillus plantarum* X, and polysaccharide from *Salvia miltiorrhiza* revealed a better beneficial effect on improving NAFLD and inhibiting LPS production than the single agent [83].

The postbiotics like exopolysaccharide from *Lactobacillus plantarum* L-14 could inhibit the differentiation of immature cells into mature adipocytes, as well as control body weight gain and lipid profiles in mice by upregulating the TLR2-AMPK signaling pathway [85]. Besides, the long-chain polyphosphate from *Lactobacillus brevis* could improve intestinal inflammation and intestinal barrier function by activating the extracellular regulated protein kinases (ERK) signaling pathway [91]. Additionally, the muramyl dipeptide from bacterial cell wall was a beneficial postbiotic which could mitigate obesity-induced insulin resistance by targeting nucleotide-binding oligomerization domain-containing protein 2 (NOD2) and interferon regulatory factor 4 (IRF4) [86]. Further study showed that the interaction between postbiotics (like muropeptide) and NOD2 could improve insulin sensitization and inflammation, but the connection with NOD1 could worsen metabolic disorders [86,87].

In short, PPSP could regulate metabolic diseases by promoting the production of beneficial bacterial metabolites, such as acetate, propionate, butyrate, isovalerate, lactic acid, and palmitoylethanolamide. PPSP also restricted the production of LPS and TMAO, as well as reduced bile acid pools (Figure 1). Moreover, PPSP lead to upregulating AMPK, ERK, GPR43, IRF4, NOD2, and TLR2-mediated signaling pathways, as well as improving tryptophan-serotonin metabolic pathways while downregulating TLR4, NF-κB, NOD1, and TNF-α-related signaling pathways (Table 1).

### 3.3. The Improvement of Intestinal Barrier Function

Intestinal barrier function, also called intestinal integrity, was strongly influenced by gut microbiota [95]. The oral administration of probiotics like *Bifidobacterium longum* NK49 and *Lactobacillus plantarum* NK3 could improve obesity and osteoporosis in mice by completing intestinal barrier integrity and further modulating immune cells with reducing TNF-α expression [71]. Besides, the single administration of *Lactobacillus fermentum* MCC2759 and MCC2760 was both beneficial to diabetic rats, but the MCC2760 strain showed a slightly better improvement in the intestinal barrier and the expression of GLUT4, GLP1, and zonula occludens-1 (ZO-1) than the MCC2759 strain [72]. Moreover, *Bifidobacterium longum* PI10 was a highly potent inducer for GLP1 and interleukin-10 (IL-10), which could strengthen the intestinal barrier [70].

Prebiotics like isomaltodextrin possessed beneficial properties on chronic inflammatory-related insulin resistance by recovering intestinal barrier with reducing endotoxin level in circulation [38]. Likely, the polysaccharides from acorn and sago could reduce gut hyperpermeability and mucosal inflammatory biomarkers in obese and type 2 diabetic mice [62]. Moreover, unsaturated alginate oligosaccharides intervention improved the intestinal barrier in obese mice due to the increase of ZO-1 and occludin expressions [42]. Furthermore, the glycolipids from tilapia heads selectively increased the enrichment of intestinal barrier-related gut microbiota, such as *Akkermansia*, *Allobaculum*, *Bifidobacterium*, *Coprococcus*, *Oscillospira*, and *Prevotellaceae* in metabolic-related colitis mice, and consequently benefited the whole metabolic system [79].

An unhealthy diet could promote the growth of LPS-produced bacteria like *Enterobacteriaceae*, lead to the translocation of LPS via the impaired intestinal barrier, and further induce dyslipidemia, insulin resistance, systemic inflammation, and immune response [96,97]. An in vivo study revealed that the administration of synbiotics containing *Lactobacillus paracasei* HII01 and xylo-oligosaccharides could improve metabolic disorder and intestinal barrier in obese rats with avoiding metabolic endotoxemia and decreasing *Firmicutes* to *Bacteroidetes* ratio and *Enterobacteriaceae* abundance [81]. Moreover, synbiotics containing *Lactobacillus paracasei* N1115 and fructo-oligosaccharides could alleviate hepatic steatosis, cirrhosis progression, and intestinal barrier in mice with NAFLD, result in improving lipid profiles, fasting blood glucose, insulin, and TNF-α by upregulating p38 MAPK pathway and the expression of tight junction proteins (like occludin 1 and claudin 1) [82].

Postbiotics also have the potential to regulate gut barrier status and could improve metabolic diseases [98]. The intestinal barrier dysfunction was related to gut microbiota dysbiosis and chronic inflammation in type 2 diabetes mellitus, and the nucleotide-binding oligomerization domain-like receptors (NLRs) played a role in the inflammatory impairment [88]. The in vitro study of colonic epithelial cells showed that the over-expression of NLRC3 potentially benefited colonic epithelial barrier integrity due to the increase of TNF receptor-associated factor 6 (TRAF6)-mediated ZO-1 and occludin expression, and butyrate could improve the intestinal barrier in type 2 diabetic mice by upregulating GPR43 expression and stimulating NLCR3 in a TRAF6-dependent manner [88]. Besides, butyrate could restore intestinal barrier function in NAFLD mice with increasing ZO-1 expression in the small intestine and decreasing the levels of gut endotoxin in serum and liver [68,90]. Another in vivo study revealed that diet supplementation with butyrate mitigated metabolic disorder and intestinal epithelial impairment in type 2 diabetic mice by promoting the secretion of insulin without compensatory hyperplasia in pancreatic β cells [89]. It should be pointed out that gut microbiota and their metabolites could drive the development of insulin resistance in obesity and T2D, possibly by initiating an inflammatory response, which has been well reviewed by a recently published paper [50].

In summary, PPSP could alleviate metabolic diseases by improving intestinal barrier function with upregulating GLUT4, GPR43, NLCR3, p38 MAPK, TRAF6-mediated signaling pathways and the expressions of claudin 1, GLP1, IL-10, occludin 1, and ZO-1 (Figure 1). PPSP could also regulate *Allobaculum*, *Akkermansia*, *Bifidobacterium*, *Coprococcus*, *Enterobacteria*, *Lactobacilli*, *Oscillospira*, and *Prevotellaceae* abundances, as well as reduce TNF-α expression and circulation endotoxin (Table 1).

## 4. Clinical Effects of Probiotics, Prebiotics, Synbiotics, and Postbiotics on Metabolic Diseases

Several clinical trials have focused on verifying the effects of PPSP on metabolic diseases, which would be discussed below.

### 4.1. Obesity

A 45-day clinical trial on 51 obese patients showed that the daily intake of fermented milk containing 2.72 × 10^10^ CFU of *Bifidobacterium lactis* could improve blood lipids and inflammatory biomarkers like TNF-α and IL-6 [99]. Moreover, a 3-week intake of probiotics containing 9 probiotic strains included *Bifidobacterium animalis* SGB06, *Bifidobacterium bifidum* SGB02, *Lactobacillus acidophilus* SGL11, *Lactobacillus delbrueckii* DSM 20081, *Lactococcus lactis* SGLc01, *Lactobacillus plantarum* SGL07, *Lactobacillus reuteri* SGL01, *Streptococcus thermophiles*, and *Streptococcus thermophilus* (1.5 × 10^10^ colony-forming units (CFU) for each) could modulate body composition, gut bacterial composition, and psychopathological status among 60 obese and pre-obese women [100]. In addition, the identification of gut microbial enterotypes could be a crucial factor for obesity management because the prevalence of obesity is lower in people with *Bacteroides* or *Prevotella* enterotypes [101]. Furthermore, the consumption of probiotics containing *Bifidobacterium breve* CBT BR3 and *Lactobacillus plantarum* CBT LP3 could more effectively improve clinical biomarkers among obese patients with the *Prevotella*-rich enterotype than the *Bacteroides*-rich enterotype [102]. Additionally, a systematic review of 20 meta-analyses involving 16,676 adults showed a moderate effect of probiotics on body weight in overweight/obesity, and the authors also point out that because these products could not be without side effects for all persons, the risk-benefit assessment should be done before their prescription [103]. In fact, some side effects of probiotics have been reported, for example, *Bifidobacterium* and *Lactobacillus* could cause infections in extremely rare cases for pregnant women and neonate because the subjects are immunocompromised, and one obese patient had mild dyspepsia and diarrhea during a 12-week probiotic intervention [102]. It should be pointed out that diet is the most important factor shaping gut microbiota, and the effect of probiotics intervention is also affected by many other factors, such as unique host and microbiome features [104]. In the further, we will enter an era of precision medicine, and will require the development of new personalized probiotic approaches.

The intake of prebiotic foods has been recognized as a potential treatment for obesity [105]. The clinical trials showed that the daily intake of yacon (a natural source of phenolic compounds and fructo-oligosaccharides) at a dose of 25 g for 6 weeks could increase the plasma antioxidant capacity, while decrease oxidative stress and fecal SCFAs levels in obese patients with no severe side effects [106,107]. Moreover, a clinical trial on 42 overweight and obese children showed that the consumption of oligofructose-enriched inulin at doses of 8 g/day for 16 weeks reduced energy intake by regulating appetite [108]. Furthermore, the daily intake of 21 g oligofructose for 12 weeks could ameliorate metabolic endotoxemia and decrease the level of plasminogen activator inhibitor 1 (PAI1) among 37 obesity patients [109]. Additionally, a 12-week clinical trial with 125 overweight or obese adults showed that both inulin-type fructan and whey protein could benefit appetite management but just fructan increased *Bifidobacterium* abundance [110].

The growing up milk is fortified milk with extra synbiotics specifically designed for children aged from 1 to 2 years old, and it could decrease the percentage of body fat with 160 children after 12 months of consumption [111]. Besides, the intake of synbiotics containing 7 freeze-dried strains (*Bifidobacterium breve*, *Bifidobacterium longum*, *Lactobacillus acidophilus*, *Lactobacillus bulgaricus*, *Lactobacillus casei*, *Lactobacillus rhamnosus*, and *Streptococcus thermophiles*, 10^9^ CFU/g for each) and 35 mg fructo-oligosaccharides could improve serum insulin level and insulin resistance with 76 obese breast cancer survivors [112,113]. Additionally, the anti-obesity effects of synbiotics were inconsistent in some trials [114,115]. A clinical trial with 59 obesity patients reported that the daily intake of 500 mg synbiotics containing *Bifidobacterium bifidum*, *Lactobacillus acidophilus*, *Lactobacillus casei* (2 × 10^9^ CFU/g for each) and 0.8 g inulin for 8 weeks improved lipid profiles and psychological status, without benefiting body mass index (BMI), blood pressure, glucose homeostasis, and waist circumference [114]. Moreover, the *Bifidobacterium adolescentis* IVS-1, the *Bifidobacterium lactis* BB-12, and the galacto-oligosaccharides showed no synergistic effects in obese patients [115]. Furthermore, a meta-analysis showed that the supplementation of synbiotics revealed no significant effects on body weight and body fat [116].

The improvement of obese patients is often accompanied by the regulation of gut microbial metabolites [117]. The intake of *Bifidobacterium lactis* UBBLa-70 and fructo-oligosaccharide for 8 weeks could increase bacterial-related metabolites like pyruvate and alanine in circulation in 32 obese women, while decrease the levels of citrate and branched-chain amino acids [118]. Further evidence implied that butyric acid possessed the potential capability to be a supportive agent in the prevention of obesity due to the properties of regulating circulation and nervous systems, as well as improving intestinal barrier, carbohydrate metabolism, immunomodulation, and appetite control [119].

In short, the *Lactobacillus* and *Bifidobacterium* strains are the most concerning probiotics and the combination of multiple probiotics possess better applied prospect. Moreover, the *Prevotella*-rich enterotype people are more sensitive to probiotics treatment than the *Bacteroides*-rich enterotype. However, the synergistic effects of probiotics and prebiotics, as well as the exact biological effects of postbiotics remain uncertain. So, the optimized formulas of synbiotics and more clinical trials for postbiotics are still needed (Table 2).

### 4.2. Type 2 and Gestational Diabetes Mellitus

A clinical trial with 50 patients with type 2 diabetes mellitus showed that the daily intake of 120 g fermented milk containing *Bifidobacterium lactis* BB-12 and *Lactobacillus acidophilus* La-5 (10^9^ CFU for each) for 6 weeks improved serum levels of fructosamine, hemoglobin A1c (HbA1c), and IL-10 [120]. Moreover, both *Lactobacillus reuteri* ADR-1 and ADR-3 strain could decrease the levels of serum HbA1c and cholesterol with regulating *Bacteroidetes* and *Bifidobacterium* abundances in 68 patients with type 2 diabetes mellitus, and the ADR-3 strain revealed a better effect on decreasing blood pressure and *Firmicutes* abundance than the ADR-1 strain [121]. Moreover, the intake of fermented milk processed by *Lactobacillus casei* Shirota for 16 weeks restricted the translocation of gut bacteria into blood circulation in 70 patients with type 2 diabetes mellitus, leading to the increased abundances of *Clostridium coccoides*, *Clostridium leptum*, and *Lactobacillus* in fecal [122]. Furthermore, several meta-analyses showed the benefits of probiotics treatment on improving HbA1c and fasting insulin [142,143]. 

The effects of probiotics on gestational diabetes mellitus remained controversial [144]. The clinical trials on pregnant women with normal body weight reported that the daily supplementation of *Lactobacillus rhamnosus* HN001 (6 × 10^9^ CFU) could lower the risk and relapse prevalence of diabetes mellitus, whereas the probiotics containing *Bifidobacterium lactis* and *Lactobacillus rhamnosus* did not prevent gestational diabetes mellitus in pregnant women with overweight or obesity [123,124]. Further evidence from 230 pregnant women with obesity revealed that the daily intake of probiotics containing *Bifidobacterium lactis* BB12 and *Lactobacillus rhamnosus* GG (6.5 × 10^9^ CFU for each) for 36 weeks did not improve depression, anxiety, and physical well-being status during pregnancy [126]. So, the bodyweight condition might be the critical factor for the effects of probiotics on gestational diabetes mellitus [125].

Prebiotics also exerted a potential in the prevention of type 2 diabetes mellitus by modulating gut microbiota dysbiosis [145,146]. A randomized placebo-controlled trial on 46 patients with type 2 diabetes mellitus showed that the intake of 10 g/day oligofructose-enriched inulin for 2 months improved glycemic status, lipid profiles, and immune biomarkers [129]. Besides, the effects of synbiotic supplementation on patients with type 2 diabetes mellitus attracted more and more attention, and the *Coix lacryma-jobi* could enhance the effect of probiotic yogurt in reducing body weight and fasting blood glucose [133]. Besides, another clinical trial indicated that synbiotics intervention just revealed a limited benefit in diabetic-related biomarkers without improving glucose metabolism [134]. Moreover, a 9-week intake of 500 mg/day synbiotics containing *Bifidobacterium*, *Lactobacillus*, *Streptococcus thermophilus*, and fructo-oligosaccharide only improved the HbA1c, BMI, and microalbuminuria in 70 patients with type 2 diabetes mellitus, without changing fasting blood glucose, lipid profiles, and creatinine [135]. Furthermore, a clinical trial compared the effects between probiotics (*Bifidobacterium bifidum*, *Bifidobacterium lactis*, *Bifidobacterium longum*, and *Lactobacillus acidophilus*, 1.5 × 10^9^ CFU for each) and synbiotics (the probiotics plus inulin) on 120 prediabetic adults, turned out that a 6-month intake of synbiotics failed to regulate gut microbial composition [136].

In general, probiotics alleviated type 2 diabetes mellitus, but the clinical application of probiotics in gestational diabetes mellitus with obesity needs further investigation. Besides, the application of bacterial-processed prebiotics is an emerging trend in clinical exploration, and the optimal formula of synbiotics as well as the specific efficacy of postbiotics on diabetes mellitus still needs more tests.

### 4.3. Other Metabolic Diseases

Metabolic impairment is a common complication of nervous system dysfunction like Alzheimer’s disease and depression [147,148]. A clinical trial on 60 patients with Alzheimer’s disease showed that a 12-week intake of probiotic milk containing *Bifidobacterium bifidum*, *Lactobacillus acidophilus*, *Lactobacillus casei*, and *Lactobacillus fermentum* at a dose of 200 mL/day (2 × 10^9^ CFU/g for each) improved cognitive function and metabolic status [127]. Likely, an 8-week consumption of probiotics (*Bifidobacterium bifidum*, *Lactobacillus acidophilus*, and *Lactobacillus casei*, 2 × 10^9^ CFU/g for each) improved metabolic status with 40 patients with major depressive disorders [128].

Prebiotics could also benefit obesity-related major depressive disorder, and the patient who received an additional 10 g/day of inulin showed a better improvement in fat mass and TC level compared to the patient who received a calorie-restricted diet alone [130]. Besides, a double-blind, placebo-control clinical trial with 75 NAFLD patients showed that the consumption of prebiotic inulin at doses of 10 g/day for 3 months could improve the grade of fatty liver and the serum levels of aminotransferase enzymes [131].

Synbiotics supplement is a promising way for controlling the prevalence of NAFLD and nervous system impairment. For example, the *Bifidobacterium* strains were widely used in different formulas of synbiotics, and the daily intake of synbiotics containing *Bifidobacterium lactis*, *Lactobacillus acidophilus*, and *Lactobacillus casei* (7 × 10^9^ CFU for each) and chicory inulin (100 mg) for 4 months could improve fatty liver grade, inflammatory and antioxidative status in 28 children with obesity-related NAFLD [138]. Moreover, a clinical trial on 79 patients with Alzheimer’s disease revealed that the daily consumption of selenium (200 mg) accompanied with *Bifidobacterium bifidum*, *Bifidobacterium longum*, and *Lactobacillus acidophilus* (2 × 10^9^ CFU for each) for 12 weeks could more effectively improve the cognitive function and metabolic function than the single selenium treatment [139]. Furthermore, the combination of *Bifidobacterium lactis* (5 × 10^9^ CFU/bag) and fructo-oligosaccharides (4.95 g/bag) could improve gastrointestinal discomfort, with decreasing the plasma levels of IL-6, IL-8, IL-17α, and interferon-γ (IFN-γ) [137]. Besides, a meta-analysis showed that the co-treatment of probiotic strains (like *Bifidobacterium* and *Lactobacillus*) and prebiotic food (like cheese) revealed the potential to be an effective intervention for metabolic syndrome [149]. 

Patients with inflammatory bowel disease were often accompanied with metabolic disorders and the depletion of butyrate-producing bacteria, and the oral intake of butyrate could enhance the efficacy of regular therapy with decreasing the *Bacteroides fragilis* to *Faecalibacterium prausnitzii* ratio [150]. Moreover, hypertension was a common complication of type 2 diabetes mellitus, and the patients who received antihypertensive medications over 3 months showed an increase in butyrate level and a decrease in acetate level, with reducing the levels of circulating fibroblast growth factor 21 (FGF 21), tumor necrosis factor superfamily member 14 (TNFSF 14) and TNF-α [151]. Furthermore, sarcopenia is a complication of the elderly diabetic population, and the utilization of butyrate-related derivatives like hydroxyl-methyl butyrate revealed a promising potential in mitigating sarcopenia [140]. In addition, bacillary dysentery is caused by the infection of *Shigella* germ, which leads to the destruction of the colonic mucosa barrier and metabolic disorders, and the adjunct therapy with butyrate could correct colonic impairment and promote antimicrobial peptides release [141].

In summary, PPSP reveal beneficial effects on metabolic-related diseases like nervous system dysfunction, hypertension, and NAFLD, meanwhile, most of the synbiotics formulas are based on *Bifidobacterium* and *Lactobacillus*. Besides, butyrate and related derivates are the most focused postbiotics but still need more clinical assessment.

## 5. Conclusions

Metabolic diseases are closely associated with gut microbiota dysbiosis. PPSP possess beneficial effects on controlling metabolic diseases such as obesity and type 2 diabetes mellitus by targeting gut microbiota. Since the beneficial effects of PPSP on alleviating metabolic diseases, researchers keep exploiting different compatible solutions for the best therapeutic effect and turns out that the co-intervention of multiple alive bacteria strains could be a promising way. Also, novel food processing strategies like enzyme-modified prebiotics and probiotic-fermented natural foods have been developed to enhance the beneficial effects. Moreover, the in vitro and in vivo studies reveal that the modulation of gut microbiota composition is one of the most direct mechanisms of PPSP to alleviate metabolic disease, manifesting as regulating the abundances of *Akkermansia*, *Bacteroidetes*, *Blautia*, *Bifidobacteria*, *Bifidobacterium*, *Collinsella*, *Clostridiaceae*, *Christensenellaceae*, *Desulfovibrionaceae*, *Firmicutes*, *Lachnospiraceae*, *Lactobacillus*, *Proteobacteria*, *Rikenellaceae*, *Ruminococcaceae*, and *Yersinia*. Furthermore, PPSP also indirectly ameliorate metabolic diseases by regulating gut microbial metabolites, such as acetate, propionate, butyrate, isovalerate, lactic acid, and palmitoylethanolamide, while suppressing the production of LPS and TMAO, as well as reducing the bile acid pools. Additionally, the improvement of the intestinal barrier function plays an important role in attenuating metabolic diseases, with upregulating claudin 1, GLP1, IL-10, occludin 1, and ZO-1 expressions. These mechanisms collectively improve the whole metabolic system by targeting several pivotal signaling pathways, such as upregulating Akt, AMPK, CPR1α, ERK, GPR43, NLCR3, NOD2, GLUT2/4, IRF4, p38 MAPK, PPARα, SIRT1, TLR2, and TRAF6-mediated pathways, while downregulating ACE, NF-κB, NOD1, TLR4, and TNF-α-related pathways. Furthermore, several clinical trials have showed the effects of PPSP on metabolic diseases, and more researches are needed on the situation when patients are during pregnancy. In addition, the optimized formula of synbiotics and the specific efficacy of postbiotics on humans are worthy of further exploration. Besides, the recent researches are mostly focusing on the effects and mechanisms of PPSP on obesity and type 2 diabetes mellitus. In the future, more attention should be paid to other metabolic diseases, such as cardiovascular diseases and hyperuricemia.

## Figures and Tables

**Table 1:** The effects and mechanisms of probiotics, prebiotics, synbiotics, and postbiotics on metabolic diseases from experimental studies.

Supplements	Models	Doses	Target Disease	Main Effects and Mechanisms	Ref.
**Probiotics**					
*Bifidobacterium lactis* LMG P-28149, and *Lactobacillus rhamnosus* LMG S-28148	in vitro, in vivo	10^8^ CFU	Obesity and insulin resistance	Restoring *Akkermansia muciniphila* and *Rikenellaceae*. Upregulating PPARγ and lipoprotein lipase expression. Enhancing insulin sensitivity and TG clearance Decreasing *Lactobacillaceae*.	[47]
*Bifidobacterium longum* PI10 and *Ligilactobacillus salivarius* PI2	in vitro, in vivo	5 × 10^8^ CFU	Obesity	Upregulating GLP1 and IL-10 expression.	[70]
*Lactobacillus plantarum* NK3 and *Bifidobacterium longum* NK49	in vivo	1 × 10^9^ CFU	Obesity and osteoporosis	Improving intestinal barrier. Suppressing LPS production. Downregulating NF-κB-linked TNF-α expression.	[71]
*Lactobacillus fermentum* MCC2760	in vivo	10^9^ CFU	Type 2 diabetes mellitus	Improving intestinal barrier. Upregulating GLUT4, GLP1, and ZO-1 expression.	[72]
*Lactobacillus casei*	in vivo	2 × 10^8^ CFU	Hypertension	Increasing *Akkermansia* and *Lactobacillus*. Decreasing *Firmicutes* to *Bacteroidetes* ratio and ACE expression.	[51]
*Bifidobacterium breve* CECT7263 and *Lactobacillus fermentum* CECT5716	in vivo	10^9^ CFU	Hypertension	Increasing butyrate-related bacteria. Elevating the plasma level of butyrate. Reducing LPS production.	[73]
*Lactobacillus johnsonii*, and *Lactobacillus reuteri*	in vivo	10^10^ CFU	Inflammatory bowel disease and Osteoporosis	Improving tetrathionate metabolism. Decreasing *Yersinia enterocolitica*.	[49]
*Lactobacillus paracasei*	in vivo	0.8–1.2 × 10^9^ CFU	Colitis with metabolic disorder	Producing palmitoylethanolamide to maintain intestinal function.	[74]
*Lactobacillus reuteri* LR6	in vivo	1 × 10^9^ CFU	Protein-energy malnutrition	Increasing *Bifidobacteria*, *Firmicutes*, and *Lactobacilli*.	[52]
**Prebiotics**					
Maize starch dextrin and Lentil	in vitro in vivo	70.8% red lentil diet	Obesity	Increasing *Actinobacteria* and *Bacteroidetes*. Decreasing *Firmicutes*.	[53,54]
Inulin oligofructose	in vivo	5% in diet, 0.6 g/day	Obesity	Increasing *Bifidobacterium*. Decreasing *Firmicutes* to *Bacteroidetes* ratio.	[58,59]
Chicory oligofructose	in vivo	10% in diet	Obesity	Increasing *Bifidobacterium* and *Collinsella*.	[60]
Amylosucrase-modified chestnut starch	in vivo	1500 mg/kg	Obesity	Upregulating SCFAs-GPR43-mediated pathway.	[23]
Fuji FF	in vivo	10% in diet	Obesity	Increasing acetic, propionic, and butyric acids production.	[75]
Unsaturated alginate oligosaccharides	in vivo	400 mg/kg	Obesity	Upregulating ZO-1 and occludin expression.	[42]
Acorn and sago polysaccharides	in vitro, in vivo	1% *v*/*v*, 5% in diet	Obesity and type 2 diabetes mellitus	Reducing gut hyperpermeability and mucosal inflammatory biomarkers.	[62]
Galacto-oligosaccharides	in vivo	7% *w*/*w*	Obesity and Insulin resistance	Increasing GLP1 expression. Decreasing fecal bile acid excretion.	[76]
Resistant dextrin from wheat and corn starch	in vivo	5 g/kg	Type 2 diabetes mellitus	Increasing *Akkermansia* and *Prevotella* abundances. Upregulating IRS1-Akt-GLUT2 and SIRT1-AMPK-PPARα-CPR1α pathways.	[56]
Isomaltodextrin	in vivo	1, 2.5, 5% in drinking water	Insulin resistance	Increasing acetic and butyric acids production. Improving intestinal barrier. Reducing circulation endotoxin level.	[38]
Whole garlic	in vivo	5% in diet	Dyslipidemia	Increasing *Lachnospiraceae*. Decreasing *Prevotella*.	[57]
Fucoidan and Galacto-oligosaccharides	in vitro, in vivo	100, 800 mg/kg	Dyslipidemia	Increasing *Bacteroidetes*, *Proteobacteria*, and the bile salt hydrolase activity of *Lactobacillus casei* DM8121. Decreasing *Actinobacteria* and *Firmicutes*.	[61]
Oat and rye brans dietary fiber	in vivo	10% in diet	Metabolic disorder	Regulating bile acids and tryptophan–serotonin metabolic pathways.	[77]
*Agave salmiana* fructan	in vivo	10% in diet	Metabolic disorder	Increasing lactic acid production.	[78]
Glycolipids from tilapia heads	in vivo	30 mg/kg	Colitis with metabolic disorder	Increasing *Akkermansia*, *Allobaculum*, *Bifidobacterium*, *Coprococcus*, *Oscillospira*, and *Prevotellaceae*.	[79]
Long-chain inulin	in vivo	5% in diet	Hypertension	Decreasing the fecal levels of acetate and propionate, and the plasma level of TMAO.	[51]
**Synbiotics**					
*Bifidobacterium*, *Lactobacillus*, *Lactococcus*, *Propionibacterium* plus omega-3 fatty acids	in vivo	2.5 mL/kg	Obesity	Revealing positively synergistic effect on reducing hepatic steatosis and lipid accumulation compared to probiotics alone.	[63,64]
*Bacillus licheniformis* plus xylo-oligosaccharides	in vivo	7.5 × 10^8^ CFU/mL and 2 g/mL	Obesity	Revealing positively synergistic effect on improving body weight gain and lipid metabolism. Decreasing *Desulfovibrionaceae* and *Ruminococcaceae*	[65]
*Lactobacillus plantarum* PMO 08 plus chia seeds	in vivo	1 × 10^9^ CFU/mL and 4% in diet	Obesity	Revealing positively synergistic effect on improving obesity. Increasing *Lactobacillus plantarum*	[66]
*Bifidobacterium lactis*, *Lactobacillus paracasei* DSM 4633, plus oat b-glucan	in vivo	10^8^ CFU and 1 g/kg	Obesity	Increasing fecal acetate, propionate, and butyrate levels. Decreasing the bile acid pools.	[80]
*Lactobacillus paracasei* HII01 plus xylo-oligosaccharides	in vivo	1 × 10^8^ CFU and 10% in PBS	Obesity	Inhibiting metabolic endotoxemia. Decreasing *Firmicutes* to *Bacteroidetes* ratio and *Enterobacteriaceae*	[81]
*Lactobacillus paracasei* N1115 plus fructo-oligosaccharides	in vivo	2.2 × 10^9^ CFU/mL and 4 g/kg/day	NAFLD	Decreasing LPS production. Downregulating TLR4 and NF-κB expression. Upregulating p38 MAPK pathway and the expression of occludin 1 and claudin 1.	[82]
*Bifidobacterium bifidum* V, *Lactobacillus plantarum* X plus *Salvia miltiorrhiza* polysaccharide	in vivo	1–2 × 10^8^ CFU and 50 mg/kg	NAFLD	Alleviating hepatic steatosis and insulin resistance improvement. Decreasing LPS level.	[83]
*Clostridium butyricum* plus corn bran	in vivo	1 × 10^8^ CFU/g and 5% in diet	Intestinal impairment with metabolic disorder	Increasing the growth of acetate-produced bacteria and the production of acetate and isovalerate. Decreasing pathogen abundances.	[84]
**Postbiotics**					
Exopolysaccharide from *Lactobacillus plantarum* L-14	in vitro, in vivo	100 μM and 500 mg/kg	Obesity	Upregulating TLR2-AMPK pathway.	[85]
Muramyl dipeptide	in vivo	100 μg	Obesity	Upregulating NOD2-IRF4 pathway	[86,87]
Butyrate	in vivo	200 mg/kg	Type 2 diabetes mellitus	Upregulating GPR43-NLCR3-TRAF6 pathway.	[88]
Butyrate	in vivo	5% in diet	Type 2 diabetes mellitus	Promoting insulin secretion without impairing pancreatic beta cells.	[89]
Butyrate	in vivo	140 mM	Metabolic disorder	Increasing *Lachnospiraceae* and *Proteobacteria*. Decreasing *Clostridiaceae*.	[67]
Butyrate	in vivo	200 mg/kg	NAFLD	Increasing *Blautia*, *Christensenellaceae*, and *Lactobacillus*.	[68]
Butyrate	in vivo	0.6 g/kg	NAFLD	Increasing ZO-1 expression. Decreasing the levels of endotoxin.	[90]
Long-chain polyphosphate from *Lactobacillus brevis*	in vivo	0.05 μg/μL	Colitis with metabolic disorder	Upregulating ERK pathway.	[91]

Abbreviation: ACE, angiotensin-converting enzyme; Akt, protein kinase B; AMPK, adenosine monophosphate kinase; CPR1α, carnitine palmitoyl transferase 1α; ERK, extracellular regulated protein kinases; GLP1, glucagon-like peptide 1; GLUT2/4, glucose transporter 2; GPR43, G-protein coupled receptor; IL-10, interleukin-10; IRF4, interferon regulatory factor 4; IRS1, insulin receptor substrate 1; LPS, lipopolysaccharide; NF-κB, nuclear factor kappa B; NLCR3, nucleotide-binding oligomerization domain-like receptors 3; NOD2, nucleotide-binding oligomerization domain-containing protein 2; p38 MAPK, p38 mitogen-activated protein kinase; PPARα/γ, peroxisome proliferators activated receptor α/γ; SCFAs, short-chain fatty acids; SIRT1, sirtuin 1; TLR2/4, TG, triglycerides; Toll-like receptor 2/4; TMAO, trimethylamine-n-oxide; TNF-α, tumor necrosis factor α; TRAF6, TNF receptor-associated factor 6; ZO-1, zonula occludens-1.

**Table 2:** The clinical effects of probiotics, prebiotics, synbiotics, and postbiotics on metabolic diseases.

Supplements	Doses	Duration	Sample Size	Target Disease	Main Effects	Ref.
**Probiotics**						
*Bifidobacterium animalis* SGB06, *Bifidobacterium bifidum* SGB02, *Lactobacillus acidophilus* SGL11, *Lactobacillus delbrueckii* DSM 20081, *Lactococcus lactis* SGLc01, *Lactobacillus plantarum* SGL07, *Lactobacillus reuteri* SGL01, *Streptococcus thermophiles*, and *Streptococcus thermophilus*	1.5 × 10^10^ CFU	3 weeks	60	Obesity	Improving body composition, bacterial composition, and psychopathological status.	[100]
*Bifidobacterium lactis*	2.72 × 10^10^ CFU	45 days	45	Obesity	Improving obesity, blood lipids, and inflammatory markers such as TNF-α and IL-6.	[99]
*Bifidobacterium breve* CBT BR3 and *Lactobacillus plantarum* CBT LP3	15 × 10^10^ CFU	12 weeks	50	Obesity	More effectively improve obese biomarkers in patients with *Prevotella*-rich enterotype than *Bacteroides*-rich enterotype.	[102]
*Bifidobacterium lactis* BB-12 and *Lactobacillus acidophilus* La-5	10^9^ CFU	6 weeks	50	Type 2 diabetes mellitus	Improving fructosamine, HbA1c and IL-10 levels.	[120]
*Lactobacillus reuteri* strain ADR-1 and ADR-3	4 × 10^9^ CFU for ADR-1 2 × 10^10^ CFU for ADR-3	12 weeks	68	Type 2 diabetes mellitus	Decreasing HbA1c and cholesterol levels.	[121]
Fermented milk processed by *Lactobacillus casei* strain Shirota	80 mL	16 weeks	70	Type 2 diabetes mellitus	Increase *Clostridium coccoides*, *Clostridium leptum*, and *Lactobacillus*. Decreasing of translocated gut bacteria.	[122]
*Lactobacillus rhamnosus* HN001	6 × 10^9^ CFU	14–16 weeks’ gestation	423	Gestational diabetes mellitus	Decreasing the relapse prevalence of diabetes mellitus.	[123]
*Bifidobacterium lactis* and *Lactobacillus rhamnosus*	1 × 10^9^ CFU	16–28 weeks’ gestation	411	Gestational diabetes mellitus	Not preventing gestational diabetes mellitus.	[124]
*Bifidobacterium* and *Lactobacillus*	1 × 10^9^ CFU	24–28 weeks’ gestation	28	Gestational diabetes mellitus	Improving glucose metabolism. Not affecting weight gain.	[125]
*Bifidobacterium lactis* BB12 and *Lactobacillus rhamnosus* GG	6.5 × 10^9^ CFU	17–36 weeks’ gestation	230	Gestational diabetes mellitus	Not improving depression, anxiety, and physical well-being status.	[126]
*Bifidobacterium bifidum*, *Lactobacillus acidophilus*, *Lactobacillus casei*, and *Lactobacillus fermentum*	2 × 10^9^ CFU/g	12 weeks	60	Alzheimer’s disease with metabolic disorder	Improving cognitive function and metabolic status.	[127]
*Bifidobacterium bifidum*, *Lactobacillus acidophilus*, and *Lactobacillus casei*	2 × 10^9^ CFU/g	8 weeks	40	Major depressive with metabolic disorder	Improving insulin resistance, C-reactive protein, and total glutathione level.	[128]
**Prebiotics**						
Yacon	25 g	6 weeks	26–40	Obesity	Increasing the plasma antioxidant capacity. Decreasing oxidative stress and fecal SCFAs levels.	[106,107]
Inulin oligofructose	8 g/day	16 weeks	42	Obesity	Decreasing energy intake by modifying appetite.	[108]
Oligofructose	21 g	12 weeks	37	Obesity	Improving metabolic endotoxemia. Decreasing PAI1 level.	[109]
Inulin-type fructan and whey protein	5–8 g	12 weeks	125	Obesity	Regulating appetite. Increase *Bifidobacterium*.	[110]
Inulin oligofructose	10 g/day	2 months	46	Type 2 diabetes mellitus	Improving glycemic status, lipid profiles, and immune markers	[129]
Inulin	10 g/day	8 weeks	45	Obesity-related major depressive	Enhancing beneficial effects of calorie-restricted diet on fat mass and TC level.	[130]
Inulin	10 g/day	3 months	75	NAFLD	Improving the grade of fatty liver and the serum levels of aminotransferase enzymes.	[131]
**Synbiotics**						
Growing up milk	300 mL	12 months	160	Obesity	Improving body fat gain.	[111]
*Bifidobacterium breve*, *Bifidobacterium longum*, *Lactobacillus acidophilus*, *Lactobacillus bulgaricus*, *Lactobacillus casei*, *Lactobacillus rhamnosus*, *Streptococcus thermophiles*, plus fructo-oligosaccharides	10^9^ CFU/g and 35 mg	8 weeks	76	Obesity	Improving serum insulin level and insulin resistance	[112,113]
*Bifidobacterium bifidum*, *Lactobacillus acidophilus*, *Lactobacillus casei*, plus inulin	2 × 10^9^ CFU/g and 0.8 g	8 weeks	59	Obesity	Improving lipid profiles and psychological status. Not benefiting BMI, blood pressure, glucose metabolism, and waist circumference.	[114]
*Bifidobacterium adolescentis* IVS-1, *Bifidobacterium lactis* BB-12, plus galacto-oligosaccharides	1 × 10^9^ CFU and 5 g	3 weeks	114	Obesity	Improving intestinal barrier function as single agents. No synergistic effects.	[115]
*Lactobacillus acidophilus* PBS066, *Lactobacillus plantarum* PBS067, and *Lactobacillus reuteri* PBS072, plus inulin and fructo-oligosaccharides	1 × 10^9^ CFU and 12.97% in liquid	2 months	60	Metabolic disorder	Decreasing metabolic syndrome prevalence	[132]
Probiotic yogurt plus *Coix lacryma-jobi*	100 mL and 25 g	12 weeks	60	Type 2 diabetes mellitus	Reducing body weight and fasting blood glucose	[133]
*Bifidobacterium bifidum* W23, *Bifidobacterium lactis* W51, *Bifidobacterium lactis* W52, *Lactobacillus acidophilus* W37, *Lactobacillus casei* W56, *Lactobacillus brevis* W63, *Lactobacillus salivarius* W24, *Lactococcus lactis* W58, and *Lactococcus lactis* W19, plus galacto-oligosaccharides P11 and fructo-oligosaccharides P6	1.5 × 10^10^ CFU and 8 g	6 months	26	Type 2 diabetes mellitus	Improving hip circumference, zonulin and lipoprotein. Not affecting glucose metabolism.	[134]
*Bifidobacterium*, *Lactobacillus*, and *Streptococus thermophilus* plus fructo-oligosaccharide	500 mg/day	9 weeks	70	Type 2 diabetes mellitus	Improving HbA1c, BMI, and microalbuminuria. Not affecting fasting blood glucose, lipid profiles, and creatinine.	[135]
*Bifidobacterium bifidum*, *Bifidobacterium lactis*, *Bifidobacterium longum*, and *Lactobacillus acidophilus* plus inulin	1.5 × 10^9^ CFU and 6 g	6 months	120	Prediabetes mellitus	Not restoring the balance of gut microbiota.	[136]
*Bifidobacterium lactis* plus fructo-oligosaccharides	5 × 10^9^ CFU/bag and 4.95 g/bag	30 days	27	Digestive disorder	Improving intestinal function. Decreasing IL-6, IL-8, IL-17α, and IFN-γ levels.	[137]
*Bifidobacterium lactis*, *Lactobacillus acidophilus*, and *Lactobacillus casei* plus chicory inulin	7 × 10^9^ CFU and 100 mg	4 months	28	NAFLD	Improving fatty liver grade, and inflammatory and antioxidative status.	[138]
*Bifidobacterium bifidum*, *Bifidobacterium longum*, and *Lactobacillus acidophilus* plus selenium	2 × 10^9^ CFU and 200 mg	12 weeks	79	Alzheimer’s disease with metabolic disorder	Improving the cognitive function and metabolic function.	[139]
**Postbiotics**						
Hydroxyl-methyl butyrate	3 g	1 week	34	Diabetic-related sarcopenia	Inhibiting catabolic effect on skeletal muscle.	[140]
Butyrate	80 mM	3 days	80	Bacillary dysentery with metabolic disorder	Improving alimentary canal function. Alleviating pathological impairment of colonic mucosa barrier Enhancing antimicrobial peptides release.	[141]

Abbreviation: BMI, body mass index; HbA1c, hemoglobin A1c; IL-6/8/10/17α, interleukin-6/8/10/17α; IFN-γ, interferon-γ; PAI1, plasminogen activator inhibitor 1; SCFAs, short-chain fatty acids; TC, total cholesterol; TNF-α, tumor necrosis factor α.
